# Early combined treatment with sildenafil and adipose-derived mesenchymal stem cells preserves heart function in rat dilated cardiomyopathy

**DOI:** 10.1186/1479-5876-8-88

**Published:** 2010-09-26

**Authors:** Yu-Chun Lin, Steve Leu, Cheuk-Kwan Sun, Chia-Hung Yen, Ying-Hsien Kao, Li-Teh Chang, Tzu-Hsien Tsai, Sarah Chua, Morgan Fu, Sheung-Fat Ko, Chiung-Jen Wu, Fan-Yen Lee, Hon-Kan Yip

**Affiliations:** 1 Division of cardiology, Department of Internal Medicine, Chang Gung Memorial Hospital-Kaohsiung Medical Center, Chang Gung University College of Medicine, Kaohsiung, Taiwan; 2 Center for Translational Research in Biomedical Sciences, Chang Gung Memorial Hospital-Kaohsiung Medical Center, Chang Gung University College of Medicine, Kaohsiung, Taiwan; 3 Division of General Surgery, Department of Surgery, Chang Gung Memorial Hospital-Kaohsiung Medical Center, Chang Gung University College of Medicine, Kaohsiung, Taiwan; 4 Department of Life Science, National Pingtung University of Science and Technology, Pingtung, Taiwan; 5 Department of Medical Research, E-DA Hospital, I-Shou University, Kaohsiung, Taiwan; 6 Basic Science, Nursing Department, Meiho University, Pingtung, Taiwan; 7 Department of Radiology, Chang Gung Memorial Hospital-Kaohsiung Medical Center, Chang Gung University College of Medicine, Kaohsiung, Taiwan; 8 Division of Cardiovascular Surgery, Department of Surgery, Chang Gung Memorial Hospital-Kaohsiung Medical Center, Chang Gung University College of Medicine, Kaohsiung, Taiwan

## Abstract

**Background:**

We investigated whether early combined autologous adipose-derived mesenchymal stem cell (ADMSC) and sildenafil therapy offers an additive benefit in preserving heart function in rat dilated cardiomyopathy (DCM).

**Methods:**

Adult Lewis rats (n = 8 per group) were divided into group 1 (normal control), group 2 (saline-treated DCM rats), group 3 [2.0 × 10^6 ^ADMSC implanted into left ventricular (LV) myocardium of DCM rats], group 4 (DCM rats with sildenafil 30 mg/kg/day, orally), and group 5 (DCM rats with combined ADMSC-sildenafil). Treatment was started 1 week after DCM induction and the rats were sacrificed on day 90.

**Results:**

The results showed that mitochondrial protein expressions of connexin43 and cytochrome-C were lowest in group 2, and lower in groups 3 and 4 than in group 5 (p < 0.002). Conversely, oxidative index was highest in group 2, and also higher in groups 3 and 4 than in group 5 (p < 0.0003). The mRNA expressions of interleukin (IL)-10, Gro/IL-8, endothelial nitric oxide synthase, and Bcl-2 were lowest in group 2, and lower in groups 3 and 4 compared with group 5 (p < 0.0001). The mRNA expressions of matrix metalloproteinase-9, Bax, caspase 3, and stromal-cell derived factor-1α were highest in group 2, and higher in groups 3 and 4 than in group 5 (p < 0.0004). Apoptosis and fibrosis in LV myocardium were most prominent in group 2 and higher in groups 3 and 4 than in group 5, whereas angiogenesis and LV ejection fraction were lowest in group 2 and lower in groups 3 and 4 than in group 5 (p < 0.003).

**Conclusion:**

Early combined ADMSC/sildenafil is superior to either treatment alone in preserving LV function.

## Background

Different treatment strategies for patients with symptomatic dilated cardiomyopathy (DCM) have been extensively investigated [1-5]. Although medications including angiotensin converting enzyme inhibitors/angiotensin II type I blockers, and beta-blockers have been recognized as some of the most effective therapeutic regimes in improving left ventricular (LV) function, congestive heart failure (CHF), and long-term outcome for patients with DCM [1-3,6,7], the mortality rate of this patient population remains high. A safe and more effective therapeutic option for improving LV function and the long-term outcome of DCM patients is urgently needed.

Growing data demonstrate that cell therapy can improve cardiac function both in the rat model of acute myocardial infarction (AMI) and in patients with ischemic cardiomyopathy or following AMI [8-12]. Cell therapy, therefore, has been suggested to be a promising novel therapeutic strategy for restoration of heart function in the settings of ischemic cardiomyopathy or AMI [8-13]. However, the potential impact of cell therapy on DCM in attenuating LV remodeling and preserving LV function has not been fully investigated [13]. Additionally, before envisaging cell-based therapy for improving ischemia-related myocardial dysfunction, some unresolved issues still need to be clarified: 1) the ideal cell source for transplantation, 2) the most appropriate route of cell administration, and, 3) the best approach to achieve an optimal cellular uptake by the recipient organ, thereby attaining a functional integration of the transplanted cells and the host tissue.

As compared with embryonic stems cells and bone marrow-derived mesenchymal stem cells, adipose-derived mesenchymal stem cells (ADMSCs) have the distinct advantages of being abundant, easy to obtain with minimal invasiveness, and readily cultured to a sufficient number for autologous transplantation without ethical issue. Previous study has also demonstrated a therapeutic superiority of ADMSCs over bone marrow-derived mesenchymal stem cells in an animal model of liver injury [14]. It is, therefore, conceivable that ADMSCs would be of tremendous momentum in translational medicine for potential clinical application in patients with cardiovascular ischemic syndrome in the near future.

Sildenafil, a phosphodiesterase type-5 (PDE-5) inhibitor, has been widely utilized in the management of erectile dysfunction in men [15,16]. Consistently, experimental studies have identified abundant distribution of PDE-5 in vascular smooth muscle cells [17] that has been demonstrated to cause vasodilatation through an increase of cyclic guanosine 3', 5'-monophospahte (cGMP) concentration [18,19]. In addition, a small clinical trial has recently reported an improvement in the symptoms of CHF in patients with DCM [20].

Although our recent study has shown that implantation of bone marrow-derived MSCs can effectively preserve cardiac function in an animal model of DCM, LV dysfunction and remodeling were actually partially rather than completely reversed by this treatment strategy [13]. Importantly, based on the experience from our clinical practice, early management is always better than a delayed treatment at all stages of development of the disease. Accordingly, the experimental protocol was designed to focus on the treatment of early stages of DCM during disease initiation rather than treatment of the established condition. The purposes of this study were to test the hypothesis that early combined treatment with autologous ADMSC implantation into LV myocardium and oral sildenafil is superior to either autologous ADMSC transplantation or sildenafil alone in the preservation of LV function in early DCM as well as to elucidate the underlying mechanisms of biologic signaling. The model used in this study is based on the development of cardiomyopathy from autoimmune myositis elicited through the administration of porcine heart myosin plus Freund complete adjuvant which is known to induce selective DCM in male Lewis rats [21].

## Methods

### Ethics

All experimental animal procedures were approved by the Institute of Animal Care and Use Committee at our hospital and performed in accordance with the Guide for the Care and Use of Laboratory Animals (NIH publication No. 85-23, National Academy Press, Washington, DC, USA, revised 1996).

### Animal Model of DCM

Experimental procedures were performed in pathogen-free, adult male Lewis rats weighing 275-300 g (Charles River Technology, BioLASCO Taiwan Co., Ltd., Taiwan). The rats were initially randomized into five groups before isolation of ADMSCs. DCM was induced via experimental myocarditis based on previous studies [21] and our recent reports [13]. Briefly, 1 mg (0.1 mL) of porcine heart myosin (Sigma) was mixed with an equal volume of Freund complete adjuvant (Sigma) and injected into the footpad of each animal on day 1 and day 7. Five weeks after immunization, these rats served as models for heart failure due to DCM [13,21].

### Isolation of Adipose-Derived Mesenchymal Stem Cells from Rat

The 20 rats in groups 3 and 5 were anesthetized with inhalational isoflurane on day 7 prior to DCM induction. Adipose tissue surrounding the epididymis was carefully dissected and excised. Then 200-300 μL of sterile saline was added to every 0.5 g of tissue to prevent dehydration. The tissue was cut into < 1 mm^3 ^size pieces using a pair of sharp, sterile surgical scissors. Sterile saline (37°C) was added to the homogenized adipose tissue in a ratio of 3:1 (saline: adipose tissue), followed by the addition of stock collagenase solution to a final concentration of 0.5 units/mL. The centrifuge tubes with the contents were placed and secured on a Thermaline shaker and incubated with constant agitation for 60 ± 15 min at 37°C. After 40 minutes of incubation, the content was triturated with a 25 mL pipette for 2-3 min. The cells obtained were placed back to the rocker for incubation. The contents of the flask were transferred to 50 mL tubes after digestion, followed by centrifugation at 600 g, for 5 minutes at room temperature. The fat layer and saline supernatant from the tube were poured out gently in one smooth motion or removed using vacuum suction. The cell pellet thus obtained was resuspended in 40 mL saline and then centrifuged again at 600 g for 5 minutes at room temperature. After being resuspended again in 5 mL saline, the cell suspension was filtered through a 100 mm filter into a 50 mL conical tube to which 2 mL of saline was added to rinse the remaining cells through the filter. The flow-through was pipetted into a new 50 mL conical tube through a 40 mm filter. The tubes were centrifuged for a third time at 600 g for 5 minutes at room temperature. The cells were resuspended in saline. An aliquot of cell suspension was then removed for cell culture in DMEM-low glucose medium containing 10% FBS for two weeks. Approximately 2.0 × 10^6 ^ADMSCs were obtained from each rat. Flow cytometric analysis was performed for identification of cellular characteristics after cell-labeling with appropriate antibodies 30 minutes before transplantation (Table 1).

**Table 1:** Flow Cytometric Results of ADMSC Surface Markers on Days 0 and 14 Cell Culture

ADMSC surface markers	Day 0 (n = 6)	Day 14 (n = 6)	p value*
CD31+	23.1 ± 6.3	43.1 ± 15.3	0.067
KDR+	18.0 ± 9.8	44.6 ± 14.7	0.040
CD45+	20.4 ± 10.5	44.6 ± 14.5	0.034
CD27+	13.5 ± 2.8	42.5 ± 16.5	0.009
VEGF+	15.3 ± 8.3	41.6 ± 17.8	0.045
vWF+	14.7 ± 8.3	43.7 ± 18.1	0.021
c-Kit+	8.8 ± 5.1	11.8 ± 7.9	0.443
Sca-1+	1.4 ± 1.1	0.7 ± 0.5	0.283
CD29+	33.8 ± 22.7	64.6 ± 19.1	0.013
CD34+	18.6 ± 7.3	4.5 ± 3.7	0.006
CD90+	42.3 ± 12.2	54.8 ± 22.0	0.257
Troponin-I+	15.4 ± 5.6	20.6 ± 15.4	0.551

*By paired-T test.

ADMSC = adipose-derived mesenchymal stem cell; VEGF = vascular endothelial cell growth factor; vWF = von Willebrand factor.

### Randomization

Eight healthy Lewis rats served as sham controls (group 1) in this study. DCM was induced in 32 Lewis rats, including those 20 rats of ADMSC isolation which were then randomized into group 2 (saline-treated DCM), group 3 (2.0 × 10^6 ^ADMSC implanted into LV anterior wall), group 4 (sildenafil 30 mg/kg/day, orally), and group 5 (combined sildenafil and ADMSC). ADMSC transplantation and oral sildenafil were given on day 7 after DCM induction, while all the animals were sacrificed on day 90.

### Rationale of Sildenafil Dosage and Early Combined Therapy

The dosage of sildenafil in this study was according to our recent report [22]. In addition, the choice of a relatively early timing of treatment was based on our aim of evaluating the therapeutic effect of the combined regimen on early DCM.

### ADMSC Labeling and Implantation

On day 14, CM-Dil (Vybrant™ Dil cell-labeling solution, Molecular Probes, Inc.) (50 μg/ml) was added to the culture medium 30 minutes before implantation of ADMSCs. After completion of ADMSC labeling, all animals were anesthetized by chloral hydrate (35 mg/kg i.p.) and placed in a supine position on a warming pad at 37°C, followed by endotracheal intubation with positive-pressure ventilation (180 mL/min) with room air using a Small Animal Ventilator (SAR-830/A, CWE, Inc., USA). Under sterile conditions, the heart was exposed via a left thoracotomy. Using a 30-gauge needle, approximately 2 × 10^6 ^ADMSCs in 100 μl culture medium IMDM were implanted in myocardium of LV anterior wall over six different sites in groups 3 and 5, while group 2 rats received 100 μl saline over the same regions of LV. Groups 1 and 4 animals received thoracotomy only without cardiac injection. After the procedures, all animals were allowed to remain on the warming pad and recover under care.

### Functional Assessment by Echocardiography

Transthoracic echocardiography was performed in each group prior to and on day 35 and day 90 after DCM induction with the anesthetized rats in a supine position by an animal cardiologist blinded to the design of the experiment using a commercially available echocardiographic system (UF-750XT) equipped with a 8-MHz linear-array transducer for animals (FUKUDA Denshi Co. Hongo, Bunkyo-Ku, Tokyo, Japan). M-mode tracings of LV were obtained with the heart being imaged in 2-dimensional mode in short-axis at the level of the papillary muscle. Left ventricular internal dimensions [end-systolic diameter (ESD) and end-diastolic diameter (EDD)] were measured according to the American Society of Echocardiography leading-edge method using at least three consecutives cardiac cycles. The LV ejection fraction (LVEF) was calculated as follows:

$$\text{LVEF}\left(\%\right)=\left[\left({\text{LVEDD}}^{\text{3}}-{\text{LVEDS}}^{\text{3}}\right)/{\text{LVEDD}}^{\text{3}}\right]\times \text{1}00$$

### Histological and Immunohistochemical Studies

Engraftment of troponin I-positive, CD31-positive, and α-smooth muscle actin (α-SMA)-positive ADMSCs was assessed by examining the implanted areas after immunohistochemical labeling using respective primary antibodies based on our recent study [13]. Irrelevant antibodies were used as controls.

### TUNEL Assay for Apoptotic Nuclei

For each rat, 6 sections (3 longitudinal and 3 transverse sections of LV myocardium) were analyzed by an in situ Cell Death Detection Kit, AP (Roche) according to the manufacturer's guidelines. The TUNEL-positive cells were examined in 3 randomly chosen high-power fields (HPFs) (×400). The mean number per HPF for each animal was then determined by summation of all numbers divided by 18.

### Integrated Area of CD31-Positively stained cells

The integrated area (μm^2^) of CD31+ spot area in the tissue sections was calculated using Image Tool 3 (IT3) image analysis software (University of Texas, Health Science Center, San Antonio, UTHSCSA; Image Tool for Windows, Version 3.0, USA) as described previously [13]. Three selected sections were quantified for each animal. Three randomly selected HPFs (400 ×) were analyzed in each section. After determining the number of pixels in each CD31+ spot area per HPF, the numbers of pixels obtained from the three HPFs were summated. The procedure was repeated in two other sections for each animal. The mean pixel number per HPF for each animal was then determined by summating all pixel numbers and dividing by 9. The mean area of CD31+ spot area per HPF was obtained using a conversion factor of 19.24 (1 μm^2 ^represented 19.24 pixels).

### Histological Study of Fibrosis Area

Masson's trichrome staining was used for studying fibrosis of LV myocardium. The method of calculating the integrated area (μm^2^) of fibrosis in LV myocardium in the tissue sections was identical to that for the integrated area (μm^2^) of CD31+ spot area using Image Tool 3 (IT3) image analysis software.

### Western Blot Analysis for Connexin (Cx)43, Cytochrome-C in Mitochondria and Oxidative Stress Reaction in LV Myocardium

Equal amounts (10-30 mg) of protein extracts from remote viable LV myocardium were loaded and separated by SDS-PAGE using 8-10% acrylamide gradients. Following electrophoresis, the separated proteins were transferred electrophoretically to a polyvinylidene difluoride (PVDF) membrane (Amersham Biosciences). Nonspecific proteins were blocked by incubating the membrane in blocking buffer (5% nonfat dry milk in T-TBS containing 0.05% Tween 20) overnight. The membranes were incubated with the indicated primary antibodies (Cx43, 1:1000, Chemicon; Cytochrome C, 1:1000, BD Biosciences; Actin, 1:10000, Chemicon) for 1 h at room temperature. Horseradish peroxidase-conjugated anti-mouse immunoglobulin IgG (1:2000, Amersham Biosciences) was applied as the second antibody for 1 h at room temperature. The washing procedure was repeated eight times within 1 h. The Oxyblot Oxidized Protein Detection Kit was purchased from Chemicon (S7150). The oxyblot procedure was performed according to a previous study [13,22]. The procedure of 2,4-dinitrophenylhydrazine (DNPH) derivatization was carried out on 6 μg of protein for 15 minutes according to manufacturer's instructions. One-dimensional electrophoresis was carried out on 12% SDS/polyacrylamide gel after DNPH derivatization. Proteins were transferred to nitrocellulose membranes which were then incubated in the primary antibody solution (anti-DNP 1: 150) for 2 h, followed by incubation with second antibody solution (1:300) for 1 h at room temperature. The washing procedure was repeated eight times within 40 minutes. Immunoreactive bands were visualized by enhanced chemiluminescence (ECL; Amersham Biosciences) which was then exposed to Biomax L film (Kodak). For quantification, ECL signals were digitized using Labwork software (UVP). For oxyblot protein analysis, a standard control was loaded on each gel.

### Vessel Density in LV Myocardium

Immunohistochemical staining of blood vessels was performed with α-SMA (1:400) as primary antibody at room temperature for 1 h, followed by washing with PBS thrice. Ten minutes after the addition of the anti-mouse-HRP conjugated secondary antibody, the tissue sections were washed with PBS thrice. The 3,3' diaminobenzidine (DAB) ( 0.7 gm/tablet) (Sigma) was then added, followed by washing with PBS thrice after one minute. Finally, hematoxylin was added as a counter-stain for nuclei, followed by washing twice with PBS after one minute. Three sections of LV myocardium were analyzed in each rat. For quantification, three randomly selected HPFs (×100) were analyzed in each section. The mean number per HPF for each animal was then determined by summation of all numbers divided by 9.

### Real-Time Quantitative PCR Analysis

Real-time polymerase chain reaction (RT-PCR) was conducted using LightCycler TaqMan Master (Roche, Germany) in a single capillary tube according to the manufacturer's guidelines for individual component concentrations. Forward and reverse primers were each designed based on individual exon of the target gene sequence to avoid amplifying genomic DNA.

During PCR, the probe was hybridized to its complementary single-strand DNA sequence within the PCR target. As amplification occurred, the probe was degraded due to the exonuclease activity of Taq DNA polymerase, thereby separating the quencher from reporter dye during extension. During the entire amplification cycle, light emission increased exponentially. A positive result was determined by identifying the threshold cycle value at which reporter dye emission appeared above background.

### Statistical Analysis

Data were expressed as mean values (mean ± SD). Statistical analysis was adequately performed by unpaired Student *t *test or analysis of variance, followed by Tukey multiple comparison procedure. SAS statistical software for Windows version 8.2 was utilized (SAS institute, Cary, NC). A probability value <0.05 was considered statistically significant.

## Results

### Group Mortality Rates

No mortality was noted in group 1 (sham control) within the study period. However, two rats died in groups 2 to 5 during the procedure or less than 3 days after the procedure. Fischer exact test revealed no significant difference in mortality rates among the five groups (p = 0.666).

### Flow Cytometry Findings of Cultured ADMSC Surface Markers

Flow cytometric analysis demonstrated that cellular expressions of the surface makers for endothelial progenitor cells (EPC) (C-kit, Sca-1) were relatively low prior to cell culture and did not significantly change after 14-day culture (Table 1). Additionally, the percentage of cells positively stained for troponin, an index of myogenic-like cell marker, was also relatively low prior to cell culture and did not significantly change after 14-day culture. However, surface makers for EPCs (CD31, CD34, KDR,) and endothelial cell (VEGF, vWF) were remarkably increased after 14-day culture. Furthermore, the expressions of surface markers of mesenchymal stem cell (CD27, CD29, CD45 and CD90) were remarkably higher following 14 days of culturing.

### Body Weight, Heart Weight, Lung Weight, and Serial Echocardiographic Findings

The initial and final body weight did not differ among the five groups, whereas the final heart weight and left lung weight were significantly higher in group 2 (i.e. DCM only) than in other groups (Table 2). There was also no significant difference in initial LVEF among all groups. Additionally, the femoral arterial blood pressure did not differ among five groups on day 90 following DCM induction. However, on day 35 and day 90 following DCM induction, the LVEF was significantly reduced in group 2 compared with that in other groups, and notably lower in groups 3 (ADMSC therapy) and 4 (sildenafil therapy) than in groups 1 (normal control) and 5 (combined ADMSC and sildenafil therapy) but it did not differ between groups 1 and 5 (Table 2).

**Table 2:** Summarized Body Weight, Heart Weight, Left Lung Weight and Heart Function in Studied Animals

Variables	Group 1† (n = 8)	Group 2† (n = 8)	Group 3† (n = 8)	Group 4† (n = 8)	Group 5† (n = 8)	p value*
Initial body weight (g)	328 ± 15.9	323 ± 11.8	323 ± 12.6	317 ± 24.3	325 ± 25.8	0.792
Final body weight (g)	450.0 ± 35.7	468.9 ± 26.4	476.5 ± 25.9	446.0 ± 29.9	465.7 ± 59.0	0.498
Final left lung weight (g)	1.70^a ^± 0.09	2.01^b ^± 0.15	1.76^a ^± 0.16	1.78^a ^± 0..21	1.78^a ^± 0.08	0.0009
Final heart weight (g)	1.46^a ^± 0.11	1.80^b ^± 0.24	1.55^a,b ^± 0.24	1.51^a,b ^± 0.28	1.42^a ^± 0.26	0.023
Day 0 LVEF (%)	79.3 ± 2.0	79.3 ± 3.5	80.9 ± 2.3	79.3 ± 3.2	80.9 ± 4.7	0.744
Day-30 LVEF (%)	79.8^a ^± 2.5	72.1^b ^± 1.7	74.8^a,b ^± 3.2	75.9^a,b ^± 5.0	78.7^a ^± 4.0	0.0005
Day-90 LVEF (%)	79.1^a ^± 3.3	70.2^b ^± 2.3	74.3^a,b ^± 0.6	74.5^a,b^± 3.8	78.6^a,b ^± 3.0	< 0.0001
FASBP on day 90, mmHg	122 ± 26	116 ± 21	109 ± 19	103 ± 22	108 ± 21	0.213

*: by one-way ANOVA. Different superscript letters between the groups indicate significant difference (at 0.05 level) by Tukey's multiple comparison procedure.

†: Group 1 = normal control; Group 2 = DCM only; Group 3 = DCM plus ADMSC; Group 4 = DCM plus sildenafil; Group 5 = DCM plus combined sildenafil and ADMSC.

FABP = femoral arterial blood pressure; LVEF = left ventricular ejection fraction.

### Identification of Implanted ADMSCs and CD31+ Cells in LV Myocardium

**Figure 1:**
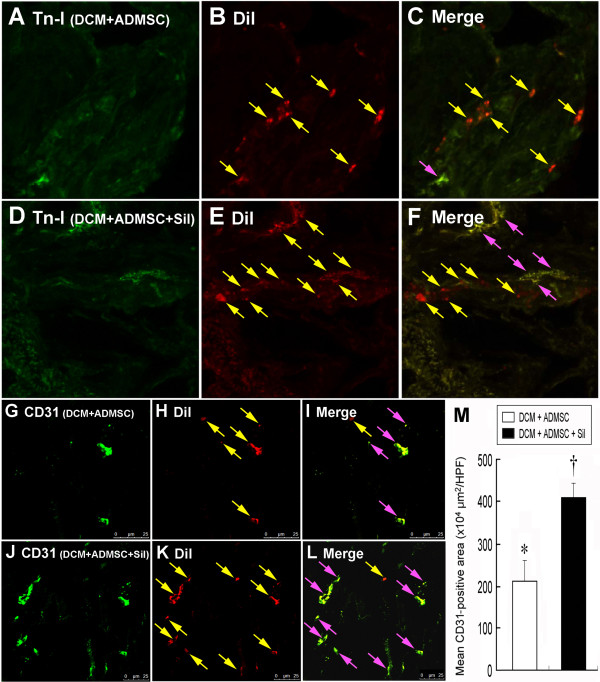
**Identification of Implanted ADMSCs and CD31+ Cells in LV Myocardium**. Confocal imaging study on day 90 following dilated cardiomyopathy (DCM induction). Merged image (C) of double staining [troponin-I (A) plus Dil (B) (yellow arrows)] in **Group 3 **(ADMSC-treated) showing few troponin I-positive myogenic-like cells (pink arrows) and undifferentiated adipose-derived mesenchymal stem cells (ADMSCs) (yellow arrows) in LV myocardium. Merged image (F) of double staining [troponin-I (D) plus Dil (E) (yellow arrows)] in **Group 5 **(combined ADMSCs and sildenafil) showing some troponin I-positive myogenic-like cells (pink arrows) and undifferentiated ADMSCs (yellow arrows) in LV myocardium. CD31-positively stained cells in **Group 3 **(G) and **Group **5 (J) indicating endothelial phenotype. Confocal image study demonstrating rich engrafting of Dil-positively stained ADMSCs (yellow arrows) in LV myocardium of **Group 3 **(H) and **Group 5 **(K). The mean CD-31 positively stain areas (pink arrows) were significantly higher (M) in **Group 5 **(L) than in **Group 3 **(I).

By day 90 following DCM induction, the rats were sacrificed for identifying implanted ADMSCs in LV myocardium. Numerous CM-Dil-stained undifferentiated ADMSCs were found to have engrafted (Figure 1B and 1E). However, only some implanted CM-Dil-stained cells presenting as myogenic-like cells were stained positively for troponin I (Figure 1C and 1F). In contrast, numerous CD31+ stained spots were identified in group 3 (Figure 1I), and significantly higher spot area (Figure 1L) was noted in group 5 as compared with group 3 in LV myocardium on day 90 after DCM induction (Figure 1M).

### Apoptosis in LV Myocardium

**Figure 2:**
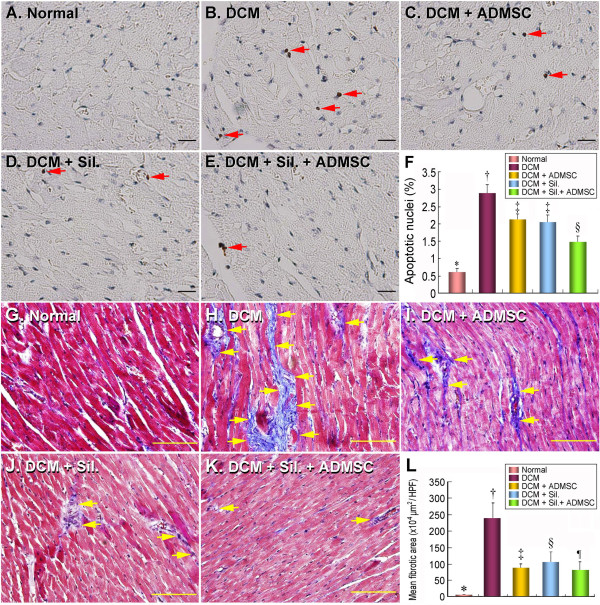
**Apoptosis and Fibrosis in LV Myocardium**. TUNEL assay (400×) of apoptotic nuclei (A-E) (red arrows) of LV myocardium on day 90 following DCM induction (n = 8). F) * p < 0.0001 between the indicated groups. Symbols (*, †, ‡) indicate significant difference (at 0.05 level) by Tukey multiple comparison procedure. Scale bars in right lower corner represent 20 μm. Mean fibrotic area (μm^2^)/high-power field (HPF) (200×) in each group (n = 8) of rats on day 90 following DCM induction. Masson's trichrome stain (G-K) demonstrating markedly increased fibrosis area (yellow arrows) in DCM group compared to other groups. L) * p < 0.001 between the indicated groups. Symbols (*, †, ‡, §, ¶) indicate significant difference (at 0.05 level) by Tukey multiple comparison procedure. Scale bars in right lower corner represent 50 μm.

The number of apoptotic nuclei was similar between groups 3 and 4 (Figure 2A-F). However, the number of apoptotic nuclei was substantially higher in group 2 than in other groups, remarkably higher in groups 3 and 4 than in groups 1 and 5, and it was also notably higher in group 5 than in group 1.

### Fibrosis of LV Myocardium

Mean area of fibrotic tissue did not differ between groups 3 and 4 on Masson's trichrome staining (Figure 2G-L). However, the mean area of fibrotic tissue was substantially higher in group 2 than in other groups, remarkably higher in groups 3 and 4 than group 5, and notably higher in group 5 than in group 1 (i.e. negative staining).

### CD40+ cell Expression in LV Myocardium and Intensity of Oxidative Stress

**Figure 3:**
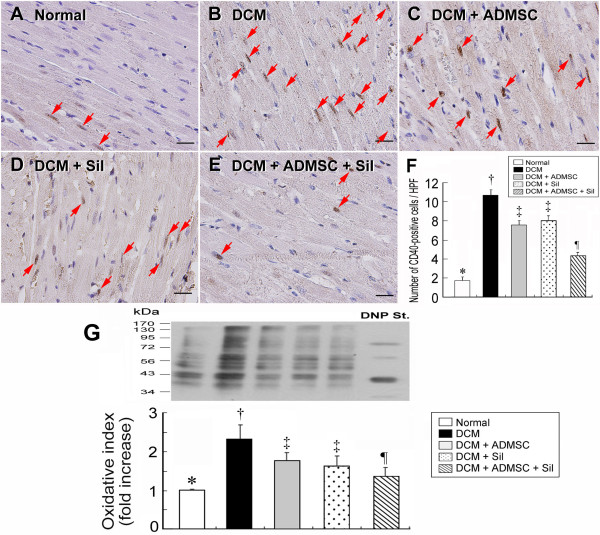
**CD40+ Cell Expression in LV Myocardium and Intensity of Oxidative Stress**. Immunohistochemical staining (400×) (A-E) for identifying CD40-positive cells (red arrows) in LV myocardium on day 90 following DCM induction (n = 8 in each group). F) * p < 0.0001 between the indicated groups. Symbols (*, †, ‡, ¶) indicate significant difference (at 0.05 level) by Tukey multiple comparison procedure. Scale bars in right lower corner represent 20 μm. Western blotting results (G) of oxidative index, protein carbonyls, in LV myocardium on day 90 following DCM induction (upper panel), with quantification results of each group (n = 8) (lower panel). * p < 0.0003 between the indicated groups. Symbols (*, †, ‡, ¶) indicate significant difference (at 0.05 level) by Tukey multiple comparison procedure. Note: Right lane and left lane shown on upper panel represent control oxidized molecular protein standard and protein molecular weight marker, respectively.

To determine whether inflammatory cells were up-regulated in LV myocardium on day 90 following DCM induction, immunohistochemical staining for detection of CD40-positively stained cells was performed (Figure 3A-F). Density of CD40-positively stained cells in LV myocardium were significantly higher in group 2 than in other groups, significantly higher in groups 3 and 4 than in groups 1 and 5, and also notably higher in group 5 than in group 1.

The oxidative stress in mitochondria did not differ between groups 1 and 5, groups 3 and 4, and groups 4 and 5 on day 90 following DCM induction (Figure 3G). However, a significantly higher mitochondrial oxidative stress was noted in group 2 than in other groups, and in groups 3 and 4 than in group 1. The oxidative stress was also notably higher in group 3 than in group 5.

### Protein Expressions of Cytochrome C and Cx43 in LV

**Figure 4:**
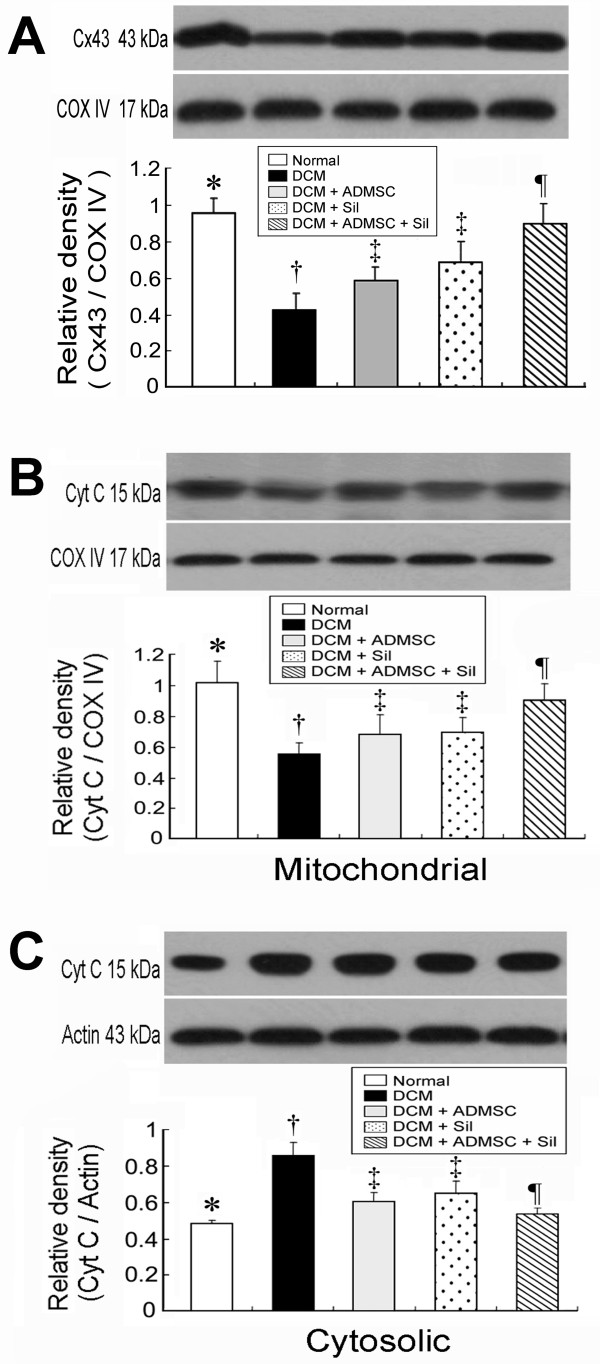
**Protein Expressions of Cytochrome C and Cx43 in LV Myocardium**. **(A) **Connexin43 protein expression of LV myocardium on day 90 after DCM induction. * p < 0.0007 between the indicated groups. **B) **Cytochrome C protein expression in mitochondria of LV myocardium on day 90 after DCM induction. * p < 0.009 between the indicated groups. **C) **Cytochrome C protein expression in cytosol of LV myocardium on day 90 after DCM induction. * p < 0.002 between the indicated groups. All symbols (*, †, ‡, ¶) in **A), B) and C) **indicate significant difference (at 0.05 level) by Tukey multiple comparison procedure (n = 8 in each group)

Western blotting for Cx43 in LV demonstrated that Cx43 protein expression was similar between groups 1 and 5 and between groups 3 and 4 (Figure 4A). However, this protein expression was substantially lower in group 2 than in other groups and notably lower in groups 3 and 4 than in groups 1 and 5 (Figure 4A).

The total amount of cytochrome C protein expression in mitochondria was similar among groups 3 and 4, and was also similar between group 1 and group 5 (Figure 4B). However, this protein expression in mitochondria was significantly lower in group 2 than in other groups, was also notably lower in groups 3 and 4 than in groups 1 and 5. The total cytochrome C protein expression in cytosol did not differ between groups 1 and 5, as well as between groups 3 and 4 (Figure 4C). However, this cytosolic protein expression was significantly higher in group 2 than in other groups, and notably higher in groups 3 and 4 than in groups 1 and 5. These findings indicate that the expression of cytochrome C, an index of energy supply and storage in mitochondria, was notably lower in group 2 than in groups 1 and 5. The increase in cytosolic cytochrome C content also suggested significant mitochondrial damage with cytochrome C release into the cytosol in the myocardium of group 2 animals.

### RT-PCR of LV Myocardium on Day 90 Following DCM Induction

**Figure 5:**
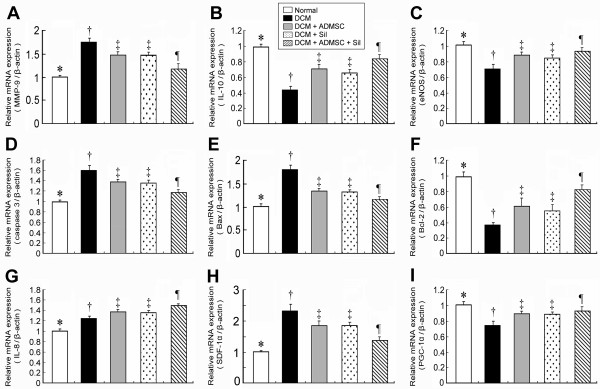
**RT-PCR of LV Myocardium on Day 90 Following DCM Induction**. The mRNA expressions of **A) **matrix metalloproteinase (MMP)-9, * p < 0.0001 between the indicated groups; **B) **interleukin (IL)-10, * p < 0.0001 between the indicated groups; **C) **endothelial nitric oxide synthase (eNOS), * < 0.0008 between the indicated groups; **D) **caspase 3, * p < 0.0004 between the indicated groups; **E) **Bax, * p < 0.0002 between the indicated groups; **F) **Bcl-2, * p < 0.0001 between the indicated groups; **G) **IL-8/Gro, * p < 0.0001 between the indicated groups; **H) **stromal cell-derived factor(SDF)-1α, * p < 0.0001 between the indicated groups; **I) **peroxisome proliferator activated receptor-γ coactivator(PGC)-1α, * p < 0.002 between the indicated groups. All symbols (*, †, ‡, ¶) in **A) to I) **indicate significant difference (at 0.05 level) by Tukey multiple comparison procedure (n = 8 in each group).

The mRNA expression of matrix metalloproteinase-9 mRNA, an indicator of inflammation, was markedly higher in group 2 than in other groups, notably higher in groups 3 and 4 than in groups 1 and 5 (Figure 5A). Conversely, interleukin (IL)-10 mRNA expression, an index of anti-inflammation, was significantly lower in group 2 than in other groups, notably lower in groups 3 and 4 than in groups 1 and 5, and significantly lower in group 5 than in group 1 (Figure 5B). Additionally, eNOS mRNA expression, an index of anti-inflammation and endothelial function, was notably lower in group 2 than in other groups, significantly lower groups 3 and 4 than in group 1 (Figure 5C). On the other hand, this mRNA expression was similar between group 1 and group 5, and was also similar among groups 3, 4, and 5.

The mRNA expressions of caspase 3 (Figure 5D) and Bax (Figure 5E), indexes of apoptosis, were remarkably higher in group 2 than in other groups, markedly higher in groups 3, 4, and 5 than in group 1, and also notably lower in groups 3 and 4 than in group 5. In contrast, mRNA expression of Bcl-2, an index of anti-apoptosis, was significantly lower in group 2 than in other groups, significantly lower in groups 3 and 4 than in than in groups 1 and 5, and also significantly lower in group 5 than in group 1 (Figure 5F). The IL-8/Gro mRNA expression, an essential chemokine guiding stem cell homing from bone marrow to damaged myocardium [23], was notably lower in groups 1 and 2 than in other groups, significantly lower in groups 3 and 4 than in group 5 (Figure 5G). Conversely, the stromal cell-derived factor (SDF) -1α mRNA expression, an index of endothelial progenitor cell chemokine attractant, was markedly increased in group 2 than in other groups, notably increased in groups 3 and 4 than in groups 1 and 5 (Figure 5H).

The peroxisome proliferator activated receptor-γ coactivator (PGC)-1α mRNA expression, an energy transcription marker, did not differ between groups 1 and 5, or among groups 3, 4, and 5. On the other hand, the mRNA expression was notably lower in group 2 than in other groups, and significantly lower in groups 3 and 4 than in group 1 (Figure 5I).

### Protein Level of eNOS, SDF-1α, Caspase 3 and Bcl-2 of LV Myocardium on Day 90 Following DCM Induction

**Figure 6:**
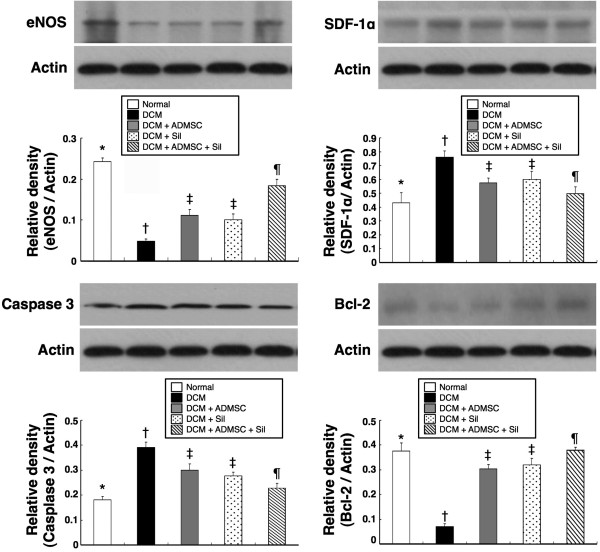
**Protein Expressions of eNOS, SDF-1α, Caspase 3 and Bcl-2 in LV Myocardium on Day 90 after DCM Induction**. **A) **eNOS protein expression. * p < 0.01 between the indicated groups. **B) **SDF-1α protein expression. * p < 0.045 between the indicated groups **C) **Caspase 3 protein expression. * p < 0.05 between the indicated groups. **D) **Bcl-2 protein expression. * p < 0.05 between the indicated groups. All symbols (*, †, ‡, ¶) in **A) to D) **indicate significant difference (at 0.05 level) by Tukey multiple comparison procedure (n = 8 in each group).

Western blot was performed to determine whether the initially elicited mRNA expressions of eNOS, SDF-1α, caspase 3 and Bcl-2 participated in translation (Figure 6). The finings showed consistent changes in protein production compared with mRNA expressions.

### Small Arteriolar Density Analysis and Cardiac Hypotrophic Gene Expression

**Figure 7:**
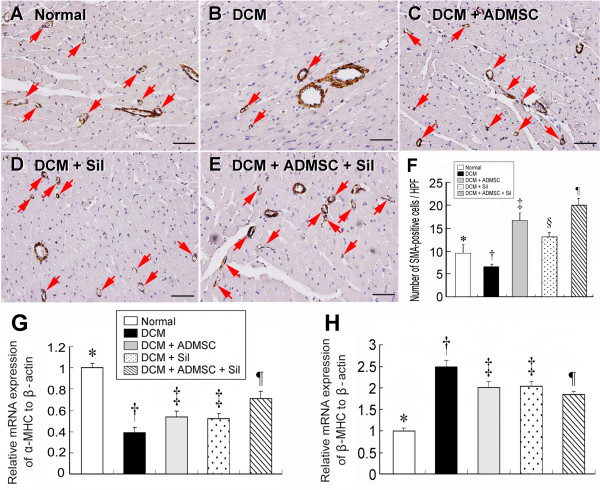
**Small Arteriolar Density Analysis and Cardiac Hypotrophic Gene Expression**. α-SMA immunohistochemical staining (A-E) (200×) for the number of small arterioles (≤ 25 μm in diameter) in LV myocardium on day 90 after DCM induction. The results showing notably lower small vessel (red arrows) number in DCM group than in other groups. F) * p < 0.0001 between the indicated groups. Scale bars in right lower corner represent 50 μm (n = 8 in each group). The mRNA expressions of α-myosin heavy chain (MHC) (G) and β-MHC of LV myocardium on day 90 after DCM induction. G) * p < 0.003 between the indicated groups. H) * p < 0.0003 between the indicated groups. All symbols (*, †, ‡, §, ¶) in **F), G) and H) **indicate significant difference (at 0.05 level) by Tukey multiple comparison procedure (n = 8 in each group).

The number of small arterioles (Figure 7A-F) (≤ 25 μm in diameter) in LV myocardium was remarkably lower in group 2 than in other groups. Moreover, the number of small arterioles was notably lower in group 1 than in groups 3, 4, and 5, significantly lower in group 4 than in groups 3 and 5, and also notably lower in group 3 than in group 5. This finding indicates that early combined treatment with sildenafil and ADMSCs is better than ADMSCs or sildenafil alone in inducing angiogenesis/vasculogenesis.

Cardiac hypertrophy is characterized by a switch of mRNA expression from α- to β-myosin heavy chain (MHC) (i.e. reactivation of fetal gene program) [24]. In the present study, the mRNA expression of β-MCH was significantly higher in group 2 than in other groups, notably higher in groups 3, 4, and 5 than in group 1, and also significantly higher in groups 3 and 4 than in group 5 (Figure 7H). No significant difference was noted, however, between group 3 and group 4. On the other hand, α-MHC in LV was expressed in a reversed manner in these groups (Figure 7G).

## Discussion

### Effect of Combined Therapy with ADMSCs and Sildenafil on Early DCM

Our recent study demonstrated an increase in heart weight and LV remodeling in the rat DCM model [13]. One interesting finding in the present study is that the left lung weight was notably higher in group 2 than in other groups on day 90 after DCM induction. This finding may implicate that an increase in left lung weight in DCM rat resulted in a sequestration of transudate due to CHF. Additionally, the heart weight was notably increased in group 2 than in groups 3 and 4, and it was remarkably increased as compared with group 5. Furthermore, RT-PCR showed substantially higher expression of the β-MHC gene in LV in group 2 than in other groups, whereas an opposite trend was noted in α-MHC gene expression in group 2 compared to other groups. Moreover, the arterial blood pressure did not differ among the five groups. These findings, in addition to supporting the reproducibility of results using our DCM model [13], further indicate that either sildenafil or ADMSC therapy offered similar effect on attenuating the progression of the hypertrophic changes in DCM that were not due to the alternation in the blood pressure. Of importance is the fact that combined therapy with ADMSCs and sildenafil is superior to either therapy alone in abrogating the progression of DCM.

### Lack of Evidence Supporting Differentiation of ADMSCs into the Myogenic-Like Cells for Preserving Heart Function

Serial echocardiographic measurements in the current study showed that LV function was significantly preserved in the DCM animals with ADMSC therapy compared with that in the DCM group without treatment on day 90 after DCM induction. Recently, we have demonstrated that bone marrow-derived mononuclear cell therapy alleviated left ventricular remodeling and improved heart function in rat DCM [13]. Accordingly, the results of our present study reinforce the findings of our recent report [13]. Interestingly, flow cytometric analysis in the present study showed only a few cells that were positively stained for troponin-I prior to or on day 14 of cell culturing. Additionally, confocal image study identified that only a few implanted ADMSCs actually differentiated into troponin-I positively stained cells in LV myocardium, a phenotype of myogenic-like cells. In contrast, a fairly large number of engrafted ADMSCs were found to exhibit the undifferentiated phenotype in LV myocardium on confocal microscopic examination on day 90 following DCM induction. These findings raise the suspicion that the number of myogenic-like cells in the LV myocardium differentiated from ADMSCs is insufficient for sustaining cardiac function. Other confounding factors, therefore, may contribute to the preservation of heart function in DCM after cellular therapy.

### Synergic Action of Early Combined Therapy with Sildenafil and ADMSCs in Preservation of LV Function in Rat DCM

Interestingly, while the effect of sildenafil on improving outcome of pulmonary arterial hypertension through enhanced vasodilatatory effect of cGMP [18,19] has been extensively investigated in both clinical trials [25] and experimental studies [23,26,27], data regarding the impact of sildenafil on improving clinical outcome of patients with DCM has been seldom reported [20]. Thus, the role of sildenafil in the DCM setting is currently unclear. An important finding in the current study was that sildenafil therapy offered similar effect compared with ADMSC therapy on preservation of heart function in DCM rats. Accordingly, our finding strengthens the finding of previous study [20]. Another important finding in the current study is that combined therapy with ADMSCs and sildenafil more significantly preserved rat LV function than either ADMSCs or sildenafil alone on days 30 and 90 after DCM induction. These findings, therefore, highlight a potential role of this combined therapy in translational clinical application in patients with DCM.

### Possible Mechanisms Underlining Improvements of Heart Function in Setting of DCM Following Cellular and Sildenafil Therapy

Recently, studies have demonstrated that angiogenesis/vasculogenesis play an essential role in improving ischemia-related LV dysfunction [10-13,25,28]. In the present study, we found that the number of small vessels and CD31-positively stained cells, an surface marker of endothelial cells, in LV myocardium were remarkably higher in DCM rats treated with ADMSCs or sildenafil than in those DCM animals without treatment, whereas it was significantly higher in the combined therapy group than in other groups. Moreover, eNOS gene and protein expressions, an index of endothelial function and angiogenesis [22], were found to show a similar increase comparable to the number of small vessels and CD31-positively stained cells in LV myocardium of each group. Interestingly, previous experimental study has demonstrated that sildenafil enhanced eNOS mRNA expression [22]. Our findings, in addition to corroborating the results of recent studies [10-13,23,25,28], may at least in part explain the mechanisms underlying sildenafil and cell therapy in preserving LV function in the rodent DCM model. Besides, our results also revealed the difference in therapeutic benefits offered by the three regimens in our experiment setting.

Growing data suggest that cytokine effect [12,13,28] is another important mechanism underlying the restoration of ischemia-related LV dysfunction. Both SDF-1α and IL-8/Gro CXC chemokines have been found to be crucial in the mobilization, incorporation, homing, survival, proliferation, and differentiation of stem cells [29,30]. In the current study, IL-8/Gro mRNA expression was found to be notably higher in animals receiving either ADMSC or sildenafil therapy and remarkably increased in the combined treatment group as compared with DCM alone. Our finding was comparable to those of previous studies [29,30]. Interestingly, mRNA and protein expressions of SDF-1α were found to show a negative correlation with that of IL-8/Gro in the animals. This finding may suggest that higher level of SDF-1 may be secreted by the ischemic tissue in response to the severity of ischemia to attract the endothelial progenitor cells for tissue repair.

The link between an increase of inflammation/reactive oxygen species (ROS) and apoptosis/cellular death in ischemic myocardium has been established [12,13,31-33]. Accordingly, our results demonstrated remarkably increased gene and protein expressions of MMP-9, the number of CD40-positively stained cells, and oxidative stress in group 2 compared with other groups. These parameters were also notably elevated in groups 3 and 4 than in group 5. In contrast, mRNA expressions of IL-10 and eNOS, the anti-inflammatory indicators, were lowest in group 2 and significantly decreased in groups 3 and 4 compared to that in group 5. Furthermore, the apoptotic biomarkers of apoptotic nuclei and Bax as well as mRNA and protein expressions of caspase 3 were notably higher, whereas both mRNA and protein expressions of Bcl-2, an anti-apoptosis biomarker, was remarkably lowest in group 2. Stem cell therapy has been proposed to be immune-modulatory and anti-inflammatory through down-regulating both innate and adaptive immunity [13,34]. Additionally, sildenafil has been found to possess anti-inflammatory, anti-fibroproliferative, and anti-apoptotic properties [23]. Our findings not only strengthen the hypothesis [13,23] and the findings of previous report [34], but also provide insight into the mechanisms underlying the reduction in fibrosis and cellular apoptosis of LV myocardium in rodent DCM after ADMSC treatment. Besides, our findings further supports that combined therapy with ADMSCs and sildenafil provide an additional benefit compared to either ADMSC or sildenafil therapy alone in significantly limiting DCM-related cardiac dysfunction.

The principal finding in the current study is that RT-PCR showed a markedly lower mRNA expressions of PGC-1α which is a transcriptional coactivator of oxidative metabolism, mitochondrial metabolism and biogenesis [13,35,36] in group 2 than in other groups, and lower in groups 3 and 4 than in group 5. Moreover, Western blot analysis identified a significantly lower mitochondrial cytochrome C content in group 2 compared with that in other groups. It was also notably lower in groups 3 and 4 than in group 5, whereas its cytosolic counterpart showed respectively opposite changes in the study groups. These findings suggest a significant preservation of mitochondrial integrity and functions from combined treatment. Furthermore, changes in Cx43 expression pattern have been reported to be associated with various cardiac pathologies and contribute to the development of cardiac arrhythmia [37]. The reduction in Cx43 protein expression in a DCM setting implies a perturbation in cell-to-cell interconnections [13] and hence electrical coupling and cellular signal transductions [37]. Of importance in the present study is that combined therapy with ADMSCs and sildenafil was better than either therapeutic option alone in preventing the down-regulation of Cx43 expression in the rodent DCM model. Therefore, not only may the current study provide explanations for the improved cardiac function after combined therapy with ADMSCs and sildenafil in the DCM animals, it also further strengthens the findings from previous studies [13,35-37].

### Study Limitation

This study has limitations. First, although sildenafil has been clearly shown to cause vasodilatation through an increase of cGMP concentration in smooth muscle [17,18], the mechanisms through which sildenafil enhanced ADMSCs' participation in the process of myocardial regeneration has not been investigated in this study. The precise role of cGMP-dependent signaling in the setting of DCM, therefore, remains unclear. Second, except for LVEF and arterial blood pressure, other physiological parameters for monitoring LV remodeling including pressure-volume loop, left ventricular end-diastolic pressure, and pulmonary vascular resistance were not provided in the current study.

**Figure 8:**
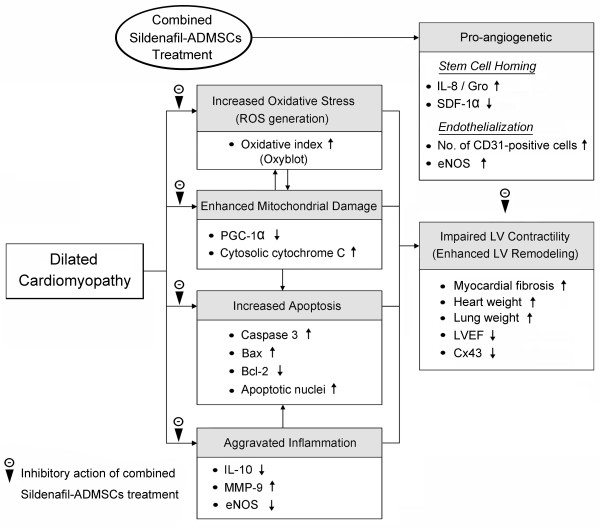
**The Proposed Mechanisms**. ADMSCs: Adipose-derived mesenchymal stem cells; ROS: Reactive oxygen species; PGC-1α: Peroxisome proliferator activated receptor-γ coactivator-1α; MMP-9: Matrix metalloproteinase-9; eNOS: Endothelial nitric oxide synthase; SDF-1α: Stromal cell-derived factor-1α; LVEF: Left ventricular ejection fraction; Cx43: Connexin 43

In conclusion, our results demonstrated that early combined treatment with ADMSCs and sildenafil for DCM rats not only is superior to either ADMSC or sildenafil alone through eliciting serial molecular-cellular biological effects in the preservation of LV function. These findings may raise the need for further prospective studies on assessing the therapeutic potential of combined ADMESC-sildenafil regimen in human subjects with DCM. The proposed mechanisms underlying the potential impacts of combined ADMSC-sildenafil therapy against DCM rats have been summarized in Figure 8.

## Competing interests

The authors declare that they have no competing interests.

## Authors' contributions

All authors have read and approved the final manuscript. Dr. Fan-Yen Lee contributed equally to this work compared with the corresponding author.

YCL, CKS, and SL designed the experiment, drafted the manuscript, and performed animal experiments. LTC, CHY, THT, SC, MF, SFK, CJW, and YHK were responsible for the laboratory assay and troubleshooting. FYL and HKY participated in refinement of experiment protocol and coordination and helped in drafting the manuscript.
